# Editorial: Neuroimaging in Parkinson's disease and Parkinsonism

**DOI:** 10.3389/fneur.2023.1153682

**Published:** 2023-02-22

**Authors:** Yu Zhang, Maria Carmela Tartaglia, Wang Zhan, Edward Ofori

**Affiliations:** ^1^War Related Illness and Injury Study Center, VA Palo Alto Health Care System, Palo Alto, CA, United States; ^2^Tanz Centre for Research in Neurodegenerative Diseases, University of Toronto, Toronto, ON, Canada; ^3^Maryland Neuroimaging Center, University of Maryland, College Park, MD, United States; ^4^Pathomechanics & Neuroimaging Laboratory, College of Health Solutions, Arizona State University, Phoenix, AZ, United States

**Keywords:** Parkinson's disease, Parkinsonism, neuroimaging, movement disorder, dopamine

As new pharmacological and surgical treatments developed, neuroimaging became one of the most promising biomarkers for diagnosis of Parkinson's Disease (PD) and Parkinsonism and monitoring disease progression and treatment. This Research Topic collected 25 articles (seven reviews, 15 original research articles, two brief research articles, and one hypothesis theory) that applied neuroimaging in PD and Parkinsonism. Imaging types included diffusion-MRI, proton-MRI, susceptibility-weighted imaging (SWI), structural and functional MRI (fMRI), glucose- and tau- positron emission tomography (PET), single photon emission computed tomography (SPECT), dopamine buffering capacity imaging, and as well as combinations of these approaches. We classified these articles into the following categories.

## 1. Meta-analysis and review articles addressing neuroimaging advantages in PD and Parkinsonism

These review articles have indicated that neuroimaging techniques are useful markers in detecting early changes in PD and parkinsonian syndromes. The brainstem and substantia nigra (SN) have been identified as vulnerable structures in PD and Parkinsonism, and neuromelanin-sensitive imaging has been shown to detect dorsolateral nigral hyperintensity, which implies SN neurodegeneration in Parkinsonism. Additionally, cognitive dysfunction is a significant non-motor feature of PD, and functional and anatomical imaging characteristics have been identified as potential predictors of cognitive impairment in PD. However, further research is required to fully understand the underlying mechanisms of cognitive impairment in PD and to develop more effective diagnostic and treatment options.

In a meta-analysis of 46 SN iron imaging studies in PD, Pyatigorskaya et al. found a notable size effect in the SN in PD for R2^*^ increase (effect size = 0.84), for SWI measurements (effect size = 1.14), and for quantitative susceptibility mapping (QSM) increase (effect size = 1.13). Correlations between imaging measurements and motor severity were mostly observed for QSM. These studies yielded consistently increased iron content in PD using R2, SWI, or QSM techniques, suggesting that these MRI measurements provide reliable markers of iron deposition in PD. Further, QSM appeared more robust and reproducible than R2^*^ and more suitable to multicenter samples.

Bergamino et al. reviewed diffusion MRI studies in early-stage PD, with a focus on recent advances in the diffusion tensor imaging (DTI) methodology, such as the advantages and disadvantages of different diffusion MRI techniques, analysis methods, and software employed. These advanced methods have been shown to detect diffuse white matter changes in early-stage PD.

Arribarat et al. proposed that the brainstem is the earliest vulnerable structure in multiple system atrophy (MSA) or PD. In a brief overview on recent advances in brainstem-related MRI markers in PD and Parkinsonism, the authors summarized the ability of brainstem imaging, including iron-sensitive MRI, nigrosome imaging, neuromelanin-sensitive MRI, DTI, and advanced diffusion imaging, to discriminate patients with PD from controls or to discriminate patients with PD from patients with atypical parkinsonism. A standardized multimodal brainstem-dedicated MRI approach is promising for detecting early changes in PD and parkinsonian syndromes.

Based on predominant protein aggregates observed within the brain, Parkinsonian disorders are categorized as a-synucleinopathies and tauopathies. Saeed et al. reviewed studies that use neuroimaging biomarkers, including structural MRI, diffusion MRI, functional MRI, proton magnetic resonance spectroscopy, and transcranial B-mode sonography, for measuring SN and lentiform nucleus echogenicity, as well as SPECT and PET that are used to improve diagnostic certainty and ensure more informed diagnostic decisions.

By reviewing multimodal MRI in Parkinsonian syndromes, Chougar et al. reported the development of several imaging signs/measurements, some of which have shown individual-level diagnostic utility. Specifically, neuromelanin-sensitive imaging detecting dorsolateral nigral hyperintensity implies SNc neurodegeneration in Parkinsonism; neuromelanin signal loss in coeruleus is associated with rapid eye movement sleep behavior disorder (RBD), and is an early sign of PD and MSA. The midbrain to pons ratio and the MR Parkinsonism Index (MRPI) are robust clinical biomarkers of progressive supranuclear palsy (PSP), while abnormalities in the putamen, pons, and cerebellum strongly support the diagnosis of MSA.

Cognitive dysfunction is a significant non-motor feature of PD; however, the pathophysiology of cognitive impairment in PD cannot be explained by dopaminergic loss alone. A review article by Sasikumar and Strafella identified functional and anatomical imaging characteristics that predict cognitive impairment in PD, the limitations that challenge this process, and the avenues of potential research.

## 2. Neuroimaging in early diagnosis of PD

Neuroimaging techniques, such as DTI, diffusion kurtosis imaging (DKI), and functional near infrared spectroscopy (fNIRS), have been found to be useful in the early diagnosis and tracking of PD. The use of DTI and DKI in particular has shown promise in identifying changes in the brain's microstructure, which may aid in diagnosing early-stage PD. Additionally, the use of fNIRS has been found to be useful in assessing changes in cortical activity during tasks such as walking, which may be indicative of compensation for executive deficits in patients with PD. Furthermore, the new assessment of dopamine transporter (DAT) using [18F]LBT-999 and short PET acquisition can be an alternative to assess dopaminergic presynaptic injury in PD using a single 10 min acquisition. These findings suggest that neuroimaging may be an important tool in the early diagnosis of PD.

Zhang and Burock reviewed DTI analyses of PD, focusing on the utility of DTI as a marker of diagnosing early PD, correlating motor symptoms, tracking disease progression, and treatment effects. This review article highlighted a complex pattern of fractional anisotropic (FA) changes in the first few years of PD, in which, FA shows compensatory responses to motor deficits in some motor areas, and these increased anisotropic findings can be observed in young-onset and early-stage PD.

Guan J. et al. evaluated mean kurtosis (MK) values in the bilateral red nucleus and SN using DKI in 26 (14 early-stage and 12 advanced-stage) patients with PD and 15 healthy controls. The authors found that MK values in the bilateral SN were significantly lower in all patients than in controls. Advanced-stage PD had a lower MK in the left SN than that of early-stage PD. While no significant differences in FA or mean diffusivity (MD) were detected between groups, the findings suggested that DKI measurements of the SN may aid the diagnoses of early PD and PD stages.

Bbingbing et al. also examined DKI and DTI in the basal ganglia nuclei and found that MD in the globus pallidus provides the largest area under the Receiver Operating Characteristic curve for distinguishing PD from healthy controls. Furthermore, all DKI parameters correlated to the Mini-Mental State Examination, and these can be a useful indicator of microstructures in extrapyramidal nuclei in PD.

Similar to the imaging of functional activity using task-based fMRI, Ranchet et al. measured cortical activity in the dorsolateral prefrontal cortex (DLPFC) by analyzing oxy-hemoglobin (1HbO2) and deoxy-hemoglobin (1HbR) using a functional near infrared spectroscopy (fNIRS) device, to asses DLPFC activation during usual walking and dual-task walking conditions in patients with PD. The authors suggested that the increased DLPFC activity in patients during usual walking suggests a potential compensation for executive deficits.

Ribeiro et al. proposed a novel assessment of DAT using [18F]LBT-999 and a short PET acquisition. The authors reported that the striatal uptake of DAT was lower than in controls, which was confirmed by binding potential, distribution volume ratios, and ratios calculated for both the striatal nuclei and SN, which were significantly lower in patients with PD compared with controls. The study demonstrated that [18F]LBT-999 PET could be an alternative to assess dopaminergic presynaptic injury in PD using a single 10 min acquisition.

## 3. Neuroimaging in monitoring PD progression and treatment-induced syndromes

Neuroimaging has played a vital role in understanding the progression and treatment-induced syndromes of PD. Studies have shown that MRI-based gray matter density can detect an organized pattern of atrophy across disease progression and that a novel pharmacological fMRI (phMRI) method can objectively quantify disease severity in PD. Additionally, pulvinar thalamic hypointensity and the putamen-to-thalamus Hyper-Perfusion/Hypo-Metabolism Index (PHI) have been proposed as potential biomarkers for cognitive worsening and Levodopa-induced dyskinesia (LID), respectively.

Blair et al. retrospectively used MRI-based gray matter density in regions of interest (ROIs) in PD studies of 228 patients with early PD, 136 patients with advanced PD, and 103 control subjects to assess the caudal-rostral pattern of the progression of Lewy body pathology in PD. The study found that patients with advanced PD had a lower gray matter density in the basal forebrain, amygdala, and entorhinal cortex than patients with early PD and controls. Across all patients with PD, the gray matter density in nearly all subcortical regions significantly decreased with disease duration. These findings suggest an organized pattern of atrophy across disease progression.

Black et al. proposed a novel phMRI method for objectively quantifying disease severity in PD. Specifically, the authors proposed a method to measure the effect site rate constant and the predicted time using phMRI, based on the timing of the known response of several brain regions to exogenous levodopa. This new method will enable measurement of the severity of dopamine denervation objectively and will simultaneously provide a robust imaging response to exogenous levodopa.

Matsuura et al. examined pulvinar thalamic hypointensity in 21 patients with PD who underwent deep brain stimulation (DBS) at baseline and 1 year after using SWI. Pulvinar hypointensity was identified in 11 of the 21 patients at baseline. One year after DBS, pulvinar hypointensity was found in five of the six patients who had worsened cognitive performance, and in all six patients who had hallucinations. These findings, i.e., pulvinar hypointensity in patients after DBS, are helpful for detecting cognitive worsening and the emergence of hallucinations.

Patients with PD and LID often present increased cerebral blood flow and decreased putaminal metabolism. Aljuaid et al. studied the effects of anti-parkinsonian medications in 10 patients with PD (5 with LID and 5 without LID), using FDG-PET and perfusion MRI in their ON and OFF states. The study found a significant interactive effect in putamen, in which glucose metabolism was consistently decreased by anti-parkinsonian medication, while mixed effects were observed in CBF perfusion. Furthermore, the putamen-to-thalamus PHI may be a potential biomarker for LID.

## 4. Neuroimaging in PD-associated motor and non-motor symptoms

Neuroimaging studies have provided new insights into the distinct characteristics of brain activity, midbrain atrophy, dysconnectivity, and metabolic alterations associated with PD. These studies have highlighted the potential of neuroimaging in detecting PD-associated motor and non-motor symptoms such as freezing of gait (FOG), depression, and cognitive dysfunction.

Li et al. evaluated motor asymmetry in PD using amplitude of low-frequency fluctuation (ALFF), assessed with resting state fMRI (rs-fMRI). The study found altered ALFF in patients with left-onset PD (LPD) compared with a control group. ALFF of the left inferior temporal gyrus correlated with motor function, and ALFF of the thalamus correlated with cognition. These findings provide new insights into the distinct characteristics of spontaneous brain activity in LPD.

Using multi-modal analyses with voxel-based morphometry and functional connectivity (FC), Droby et al. reported midbrain atrophy in patients with PD and FOG compared with those without FOG. Furthermore, decreased midbrain-cortical FC was found to be associated with FOG occurrence and the severity scores of patients with PD.

To reveal the dysconnectivity mechanism underlying PD with and without freezing of gait, Jin et al. analyzed structural and functional connectivity using FA and MD measurements from DTI and voxel-mirrored homotopic connectivity (VMHC), calculated from rs-fMRI images of 24 patients with PD and FOG+, 37 patients with PD and FOG–, and 24 healthy controls. Patients with PD and FOG+ had impaired interhemispheric brain connectivity, measured by FA, MD, and VMHC, which was related to clinical FOG severity. These results demonstrate that integrating structural and functional MRI analyses can provide information on the pathophysiological mechanisms of FOG in PD.

Wang et al. studied brain blood-oxygen-level-dependent (BOLD) activities using Regional Homogeneity (ReHo), calculated from rs-fMRI. The authors reported that mildly-moderately depressed PD had a lower ReHo in the anterior cingulate cortex than that for non-depressed PD. Severely depressed PD had a higher ReHo in the frontal area than that for mildly-moderately depressed PD. Severely depressed PD had a higher ReHo in the supplementary motor area than that for non-depressed PD. ReHo measurement may provide evidence for the prediction of PD with subclinical depression.

Guan J-t. et al. used 1H-MRS to investigate the alteration of metabolites in patients with PD at different stages of the disease. The results revealed that altered metabolites are associated with non-motor symptoms, including sleep and gastrointestinal and cognitive dysfunction in patients with PD at late stages, suggesting that these metabolic markers may be able to track disease progression in PD.

## 5. Neuroimaging in differentiating among atypical Parkinsonism

Atypical Parkinsonism shares typical motor symptoms with sporadic PD, and misdiagnosis is frequent. Measuring neuromelanin-sensitive signals as well as structural indices of the brainstem, cerebellum, midbrain, and basal ganglia nuclei help differentiate corticobasal syndrome (CBS), PSP, and MSA from PD. Tau-PET and DAT imaging have shown strong effects in differentiating dementia with Lewy bodies (DLB) from Alzheimer's disease (AD).

Picillo et al. performed midbrain-based MRI morphometric assessments in 67 patients with PSP. The study reported that reduced midbrain size is significantly associated with greater ocular motor dysfunction at baseline MRI as well as more rapid disease progression upon follow up.

Vasilevskaya et al. compared parkinsonian syndromes (CBS/PSP) without AD biomarkers (CBS/PSP-non-AD, *n* = 17) to parkinsonian syndromes with AD biomarkers (CBS/PSP-AD, *n* = 7), using MRI and PET imaging with a tau ligand [18F]-AV1451 tracer. The AD biomarker–positive group had increased Tau-uptake and lower volumes in AD-specific areas. These results suggested that CBS and PSP syndromes are strongly affected by the presence of AD biomarkers.

Talai et al. compared the accuracy of using multi-modal MRI datasets for automatic differentiation of patients with PD, patients with progressive supranuclear palsy Richardson's syndrome (PSP-RS), and healthy controls. Machine learning models showed that regional DTI metrics performed excellently, with a 95.1% classifying accuracy, similar to the optimal multi-modal machine learning model. These uni-modal DTI and multi-modal (morphometric T1WI, iron-sensitive T2WI, and DTI) datasets may provide useful markers in differentiating PD and PSP-RS.

The cingulate island sign (CIS), which is a preserved pattern in the posterior cingulate gyrus on FDG-PET, is an important sign to distinguish DLB from AD. Kanetaka et al. studied CIS using IMP-SPECT in patients with mild cognitive impairment (MCI), AD, or DLB. The CIS score in the AD group was significantly higher than that in the DLB or MCI groups. The results suggest that the diagnostic power of IMP-SPECT is not inferior to that of ECD-SPECT in differentiating DLB from AD.

Inagawa et al. investigated the efficacy of olfactory and hallucination tests in differentiating AD from DLB, in comparison with the known biomarkers for DLB, including DAT-SPECT and meta-iodobenzylguanidine (MIBG) in myocardial scintigraphy. Their results suggest that the hallucination and olfactory tests may be considered before resorting to nuclear neuroimaging in the diagnosis of DLB.

In conclusion, this Research Topic provided articles on well-established and emerging neuroimaging biomarkers to improve diagnostic certainty and to ensure more precise treatment strategies. A remarkable advantage is the integrative multimodal neuroimaging approach, which proved superior to single modality-based methods. We hope that readers will find this Research Topic a useful reference for state-of-the-art neuroimaging developments and applications in Parkinson's Disease and Parkinsonism.

## Author contributions

All authors listed have made a substantial, direct, and intellectual contribution to the work and approved it for publication.

